# The novel use of oral antibiotic monotherapy in prosthetic valve endocarditis caused by *Finegoldia magna*: a case study

**DOI:** 10.1186/s13019-019-0993-9

**Published:** 2019-09-18

**Authors:** Siobhan Chien, David Gorman, Charilaos-Panagiotis Koutsogiannidis, Ramanish Ravishankar, Ganesh Kamath, Vipin Zamvar

**Affiliations:** 1 Department of Cardiothoracic Surgery, Royal Infirmary of Edinburgh, 51 Little France Crescent, Edinburgh, EH16 4SA UK; 2 University of Edinburgh, Edinburgh, UK; 3 Department of Cardiothoracic Surgery, Kasturba Medical College, Manipal, India

**Keywords:** *Finegoldia magna*, Prosthetic valve endocarditis, Oral antibiotics, Administration, Cardiac surgical procedures, Treatment outcome, Infective endocarditis

## Abstract

**Background:**

*Finegoldia magna*, a Gram-positive anaerobic coccus, is part of the human normal microbiota as a commensal of mucocutaneous surfaces. However, it remains an uncommon pathogen in infective endocarditis, with only eight clinical cases previously reported in the literature.

Currently, infective endocarditis is routinely treated with prolonged intravenous antibiotic therapy. However, recent research has found that switching patients to oral antibiotics is non-inferior to prolonged parenteral antibiotic treatment, challenging the current guidelines for the treatment of infective endocarditis.

**Case presentation:**

This case report focuses on a 52-year-old gentleman, who presented with initially culture-negative infective endocarditis following bioprosthetic aortic valve replacement. Blood cultures later grew *Finegoldia magna*. Following initial intravenous antibiotic therapy and re-do surgical replacement of the prosthetic aortic valve, the patient was successfully switched to oral antibiotic monotherapy, an unusual strategy in the treatment of infective endocarditis inspired by the recent publication of the POET trial. He made excellent progress on an eight-week course of oral antibiotics and was successfully discharged from surgical follow-up.

**Conclusions:**

This case is the 9th reported case of *Finegoldia magna* infective endocarditis in the literature. Our case also raises the possibility of a more patient-friendly and cost-effective means of providing long-term antibiotic therapy in suitable patients with prosthetic valve endocarditis and suggests that the principles highlighted in the POET trial can also be applicable to post-operative patients after cardiac surgery.

## Background

*Finegoldia magna* (previously known as *Peptostreptococcus magnus*) is the most commonly isolated Gram-positive anaerobic cocci (GPAC) from clinical specimens, and is a normal commensal of human mucocutaneous surfaces [1, 2]. However, it remains a rare and unusual microbe implicated in the pathogenesis of infective endocarditis.

Although relatively rare, infective endocarditis carries a substantial morbidity and mortality risk, with current guidelines advocating the administration of prolonged, parenteral bactericidal therapy for complete infection eradication [3]. The recent publication of the Partial Oral Treatment of Endocarditis (POET) trial has recently proven switching patients with infective endocarditis to oral antibiotic therapy to be non-inferior to continued intravenous (IV) antibiotic therapy [4], challenging whether prolonged administration of parenteral antibiotics is necessary in a specific subset of patients with infective endocarditis.

## Case report

A 52-year-old man presented to our Emergency Department with sweats, lethargy and weakness 5 weeks following tissue aortic valve replacement with a 23 mm bioprosthetic valve. His background included severe aortic stenosis, mild left ventricular impairment, atrial fibrillation, hyperthyroidism, obstructive sleep apnoea and autistic spectrum disorder.

Following the original aortic valve replacement, the patient had made a good recovery, although he had been noted to be sweating at times. He maintained a normal white cell count and was apyrexial throughout, and was discharged home on postoperative day 6. Immediately following discharge, he did reattend via the Emergency Department with sweats and back pain, and was diagnosed with a lower respiratory tract infection.

Having re-presented with generalised weakness, lethargy and sweats, clinical examination was unremarkable and bloods demonstrated only a raised C-reactive protein (CRP) of 48. Trans-thoracic echocardiography demonstrated no evidence of endocarditis. Blood cultures taken on admission were initially reported as negative. However, during his admission the patient became pyrexial with worsening sweats and rigors. Urine culture, viral throat swab, CT scanning and dental review revealed no clear source of infection. There was no suggestion of immunocompromise or other risk factors for infection with this unusual pathogen.

**Fig. 1:**
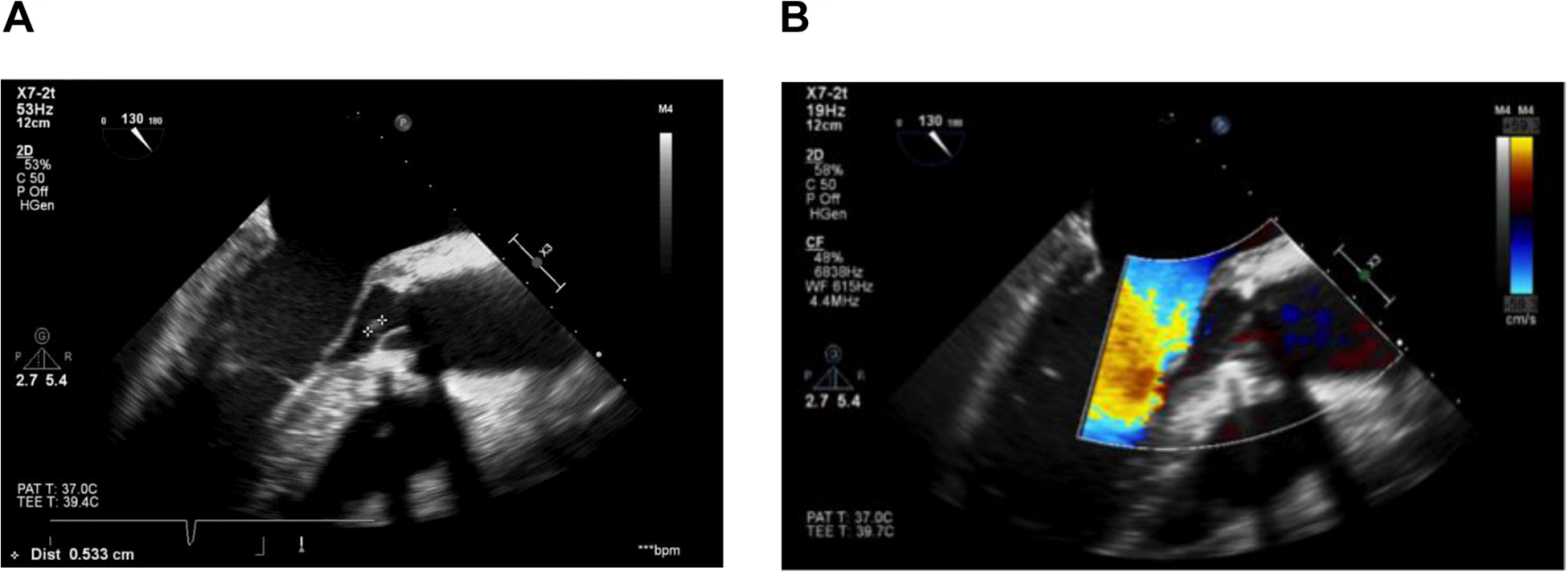
Trans-oesophageal echocardiography (TOE) showing (**a**) location and size of vegetation and (**b**) mitral regurgitation

Trans-oesophageal echocardiography on day 6 revealed infective endocarditis of the prosthetic aortic valve (see Fig. 1). Antibiotic cover was broadened to IV vancomycin, IV gentamicin and oral metronidazole. Pyrexia and inflammatory markers improved following this. IV gentamicin was later stopped on microbiology advice. Four days following admission, one set of blood cultures grew a Gram-positive anaerobe coccus, later identified as *Finegoldia magna* on day ten. Antibiotic sensitivities were assessed using gradient strip diffusion and are shown in Table 1.

**Table 1:** Antibiotic sensitivities of cultured *F. magna*. A lower MIC value indicates greater sensitivity to the tested antibiotic

Antibiotic tested	Minimum inhibitory concentration (MIC)
Clindamycin	0.064 mg/L
Co-amoxiclav	0.500 mg/L
Metronidazole	0.047 mg/L
Penicillin	0.064 mg/L
Vancomycin	0.094 mg/L

Intravenous antibiotic therapy was commenced. The decision was taken to resort to surgical intervention with replacement of the infected prosthetic valve. The patient underwent re-do surgery with removel of the infected valve and implantation of another bioprosthetic valve. Culture and sensitivities from the excised valve were negative.

After 19 days of IV vancomycin and oral metronidazole, vancomycin was stopped. Given the excellent oral bioavailability of metronidazole, the decision was taken to treat the anaerobic infective endocarditis with oral metronidazole monotherapy. The patient’s improving clinical condition, improving inflammatory markers and the achievement of source control through surgery supported the decision to switch to oral therapy.

The patient progressed well and was discharged on post-operative day 13 to complete a total 8-week course of oral metronidazole from the date of re-do surgery. Regular monitoring of CRP confirmed continuing improvement. He was discharged from follow-up eight weeks later, with final “test of cure” blood cultures reported as negative. No adverse effects following re-operation and prolonged antibiotic treatment were reported.

## Discussion

*Finegoldia magna,* previously known as *Peptostreptococcus magnus* [5], is a Gram-positive anaerobic coccus (GPAC), and like other GPACs is a common commensal of human skin, mucous membranes, and gastrointestinal and genitourinary tracts [6–8].

Of the GPACs, *F. magna* is thought to be amongst the most pathogenic, and is a common isolate from skin and soft tissue infections, bone and joint infections, and diabetic foot infections [7, 9, 10]. Its particular virulence is thought to be the product of a number of adaptations. Well described are: the expression of pili, which assist in adhesion and colonisation; the ability to bind to human albumin by way of a peptostreptococcal albumin-binding protein leading to accelerated growth rates; and expression of superantigens targeting B cells (the most common being Protein L) [7, 11, 12].

Whilst the infections above due to *F. magna* appear to be relatively common, confirmed infective endocarditis due to *F. magna* is notably more rare. A search of the English-language literature reveals eight previously reported cases of endocarditis with *F. magna* or *P. magnus* as the confirmed causative organism.

Of note, blood cultures were negative in the majority of *F. magna* infective endocarditis cases, with definitive diagnosis made only by tissue specimens obtained during surgery [13]. Blood cultures yielded a positive microbiology result after four days in our case. Culture-negative infective endocarditis has a potentially negative impact on patient management, as this can lead to delays in the diagnosis and the initiation of the appropriate antibiotic regimen according to sensitivities [14]. Therefore, it is important to consider *F. magna* infection in a patient presenting with clinical suspicion of infective endocarditis after cardiac valvular surgery with initially sterile blood cultures [13].

According to guidelines from the European Society of Cardiology (ESC) and the American Heart Association (AHA), management of infective endocarditis classically involves the administration of intravenous antibiotics for up to 6 weeks’ duration [3, 15]. The majority of complications, including in-hospital mortality, are found to occur within the initial phase after admission [16–18]. A significant number of clinically stable patients are therefore subjected to a prolonged hospital admission after this initial phase to facilitate the administration of intravenous antibiotic therapy. Prolonged hospital stay is associated with an increased physical and psychological burden [19–22], whereas shorter inpatient admissions have better outcomes in other diseases and are more cost-effective [23–25]. The European and American guidelines consequently recommend the use of outpatient parenteral treatment in patients fulfilling certain criteria to alleviate the risks and complications associated with longer duration of hospital stay [3, 15, 26, 27].

However, the use of outpatient parenteral treatment is not without its challenges. For example, logistical issues must be addressed, patient and staff education is fundamental, close monitoring is required for efficacy and adverse effects, and patients must receive easy access to medical advice, in addition to adequate social and psychological support [4]. It has previously been hypothesised that the use of oral antibiotic therapy for the latter stages of the treatment period in stable patients with infective endocarditis would pose an appropriate alternative solution to these challenges, by allowing treatment to take place outside hospitals and without the need for intravenous catheters. However, this approach has been restricted due to the limited clinical evidence base for the safety and efficacy of oral antibiotic therapy for infective endocarditis [28–32].

The POET trial was published in the New England Journal of Medicine in 2018. 400 clinically stable patients with infective endocarditis on the left side of the heart were involved in this randomised unblinded trial, and were assigned to either continue intravenous treatment or to switch to oral antibiotic treatment, after the administration of at least 10 days of intravenous antibiotics. At the time of randomisation, at least 10 days of scheduled antibiotic treatment had to remain. The patients in the orally treated group were then discharged to outpatient treatment, if feasible. The POET trial confirmed that, in clinically stable patients with endocarditis on the left side of the heart, changing to oral antibiotic therapy was non-inferior to continued intravenous antibiotic therapy [4]. This supports the treatment of this patient with prolonged duration oral antibiotic therapy.

Previously, it had been suggested that intravenous antibiotic therapy was superior to the use of oral agents for infective endocarditis, with the main concern suggestive that gastric absorption of oral agents may be inadequate to allow sufficient plasma concentrations of antibiotics to facilitate bacterial killing [24]. The POET trial emphasises the importance of normal gastrointestinal function to facilitate the uptake of orally administered antibiotics [4]. The patient was commenced on IV vancomycin and oral metronidazole originally based on sensitivity results. Vancomycin must be administered via the intravenous route to adequately treat most infections, due to its poor oral bioavailability (< 10%) [33].

There is other evidence to support the choice of oral metronidazole monotherapy: oral metronidazole is known to have a high bioavailability (98.9%) [34], with similar peak serum concentrations when administered via either the oral or intravenous route [35]. Oral metronidazole tablets are therefore often the preferred mode of administration, if tolerated. In this case, metronidazole was found to have a minimum inhibitory concentration of 0.047 mg/L, approximately 1/85th of the breakpoint concentration at which an organism is deemed metronidazole-sensitive, as per EUCAST guidelines current at the time of treatment [36]. This gave a good degree of confidence that metronidazole monotherapy would provide sufficient antimicrobial therapy.

Although clindamycin was indicated as another possible therapy by sensitivity testing, it is not usual practice in the UK to use clindamycin in the treatment of any bacterial endocarditis [37]. Furthermore, it was felt that a prolonged course of clindamycin was potentially harmful due to the significant associated risk of *Clostridium difficile* infection [38]. This supported the decision to treat with oral metronidazole alone.

In contrast to our case, the POET trial includes patients with endocarditis on the left side of the heart caused by *Streptococcus*, *Enterococcus faecalis*, *Staphylococcus aureus*, or coagulase-negative *Staphylococci* [4]. In the case of this patient, blood cultures were positive for *F. magna*, an anaerobic organism. Infective endocarditis caused by anaerobic pathogens remains relatively uncommon, and accounts for only 2–16% of all cases of infective endocarditis over the past four decades [39–42]. The POET trial did not include patients in whom an anaerobic pathogen was the causative organism. This case has demonstrated that switching to oral antibiotic therapy in a patient with an anaerobic infective endocarditis could also be satisfactory, but further research is required to prove if this statement could be substantiated with clinical evidence.

In addition, when switching to the oral route in the POET trial, all oral regimens included two antibiotics from different drug classes and with different antibacterial effects and different metabolization processes. This was to reduce the risk of pharmacokinetic variations of the orally administered antibiotics [43]. We have differed in offering only metronidazole as monotherapy, for the reasons outlined above and with a good degree of confidence that it would provide suitable cover. The POET trial has demonstrated that oral therapy with two agents was sufficient in the treatment of aerobic endocarditis in selected cases [4]. We have added to this evidence a case in which an anaerobic endocarditis was found to be treatable with oral monotherapy alone. In a time of renewed focus on antibiotic stewardship and avoidance of unnecessary use of antimicrobials, this is an exciting development.

Our patient received a total of 62 days of oral metronidazole, with only 6 days of IV metronidazole given during the immediate post-operative period in the intensive care setting, compared to only 19 days of IV vancomycin. Follow-up demonstrated clinical and biochemical evidence of cure. This suggests that the use of single agent oral antibiotic regimens may be a possible development in the future of infective endocarditis treatment. However, further research is required to determine the safety and efficacy of oral antibiotic monotherapy in the future.

## Conclusion

We have discussed an unusual case of *F. magna* endocarditis affecting a bioprosthetic heart valve. Because of the tendency toward negative blood cultures in the early stages of diagnosis, a high index of suspicion is needed to diagnose endocarditis in vulnerable patients with non-specific features. This case also demonstrates successful treatment of an anaerobic endocarditis with outpatient oral antibiotic therapy. This builds on previous work which has shown outpatient oral antibiotics to be a feasible treatment for selected cases. Further research in this area may lead to practice changes that significantly reduce length of hospital stay, and its associated risks and costs, in these patients.

## Data Availability

Data sharing is not applicable to this article as no datasets were generated or analysed during the current study.

## Abbreviations

AHA: American Heart Association
AV: aortic valve
CRP: C-reactive protein
CT: computed tomography
ESC: European Society of Cardiology
GPAC: Gram-positive anaerobic cocci
IV: intravenous
MIC: minimum inhibitory concentration
MV: mitral valve
POET trial: Partial Oral Treatment of Endocarditis trial
